# Vegetarianism

**Published:** 1888-04-21

**Authors:** Richard Greene

**Affiliations:** Berry Wood.


### The Editor's Letter-Box.

[Our Correspondents are reminded that prolixity is a great bar to publication, and that brevity of style and conciseness of statement greatly facilitate early insertion.]

### VEGETARIANISM.

In common with all those who believe that the so-called Vegetarianism is incomparably the best form of diet for man I was not altogether displeased with your annotation on the subject in last week's Hospital; albeit, I think you do wrong to speak of the system as a "fad." A "fad" the dictionary tells us is a "hobby" or "craze," and the word would seem to be derived from the verb "fade," meaning to "wither" or "perish gradually." Now the Vegetarian Society was formed about forty years ago by a very few individuals, and it has since its formation enrolled 3,240 members and 1,500 associates. The society includes representatives of the aristocracy, gentry, clergy, law, medicine, literature, science, merchants, clerks, mechanics, and even such laborious occupations as blacksmiths and iron puddlers; and it is increasing not decreasing its numbers. There are upwards of forty restaurants in England, and there are at least two journals in circulation. Vegetarianism (I use the old word for want of a better) rests on science, on humanity, on experience, on morality, and on economy. On science, because it has been proved that man resembles very closely the frugivorous animals in everything and the carnivorous animals in nothing. On humanity, because no animal is sacrificed, no suffering entailed, to produce the bloodless meal of fruits. On experience, because the testimony of those who have given it a really fair trial is almost unanimous in its favour. On morality, because the vegetarian is nearly always temperate in the consumption of alcohol, and we are told on the highest authority that it is alcohol which causes "infinite sin, misery, madness, and crime." On economy, because the land of England would support five times as many people on cereals as it can do on beef; and the farmer, blessed with plenty, would no longer weep the vanished golden age. As to the practice being preventive or curative of disease it would manifestly be impossible to enter into the question in a short letter; but it has been stated (Metropolitan Inspector, quoted by Dr. A. Kingsford) that a very high percentage, as high as 86 per cent., I think, of the carcases sold for food is diseased, tuberculous disease being the chief. It is becoming a common belief among medical men that phthisis may be communicated from the inferior animals to man. (Lamallerée, Valin, McColl, &c.), and yet it is almost the invariable rule to order animal food for consumptive patients. This is the *similia similibus curantur* doctrine pushed to its extreme limits. Doubtless perfect cooking impairs the activity of disease germs, but even then the idea of eating tuberculous flesh and drinking tuberculous milk is not pleasant, and it is certain that more than one sufferer from our insular scourge dates his recovery from the day he gave up eating animal food. Apologising for taking up so much of your space, I am, &c.,

Berry Wood. Richard Greene.

